# Synthesis and Characterization of 3-(1-((3,4-Dihydroxyphenethyl)amino)ethylidene)-chroman-2,4-dione as a Potential Antitumor Agent

**DOI:** 10.1155/2019/2069250

**Published:** 2019-02-13

**Authors:** Dušan S. Dimić, Zoran S. Marković, Luciano Saso, Edina H. Avdović, Jelena R. Đorović, Isidora P. Petrović, Danijela D. Stanisavljević, Milena J. Stevanović, Ivan Potočňák, Erika Samoľová, Srećko R. Trifunović, Jasmina M. Dimitrić Marković

**Affiliations:** ^1^University of Belgrade, Faculty of Physical Chemistry, Studentski trg 12-16, 11000 Belgrade, Serbia; ^2^Department of Chemical-Technological Sciences, State University of Novi Pazar, Vuka Karadžića bb, 36300 Novi Pazar, Serbia; ^3^Department of Physiology and Pharmacology “Vittorio Erspamer”, Sapienza University of Rome, Rome, Italy; ^4^University of Kragujevac, Faculty of Science, Radoja Domanovića 12, 34000 Kragujevac, Serbia; ^5^BioIRC, Bioengineering R&D Center, Prvoslava Stojanovića 6, 34000 Kragujevac, Serbia; ^6^University of Belgrade, Institute of Molecular Genetics and Genetic Engineering, Vojvode Stepe 444a, PO Box 23, 11010 Belgrade, Serbia; ^7^University of Belgrade, Faculty of Biology, Studentski trg 16, 11000 Belgrade, Serbia; ^8^Serbian Academy of Sciences and Art, Knez Mihajlova 35, 11000 Belgrade, Serbia; ^9^Institute of Chemistry, P. J. Šafárik University in Košice, Moyzesova 11, 04154 Košice, Slovak Republic, Slovakia

## Abstract

The newly synthesized coumarin derivative with dopamine, 3-(1-((3,4-dihydroxyphenethyl)amino)ethylidene)-chroman-2,4-dione, was completely structurally characterized by X-ray crystallography. It was shown that several types of hydrogen bonds are present, which additionally stabilize the structure. The compound was tested *in vitro* against different cell lines, healthy human keratinocyte HaCaT, cervical squamous cell carcinoma SiHa, breast carcinoma MCF7, and hepatocellular carcinoma HepG2. Compared to control, the new derivate showed a stronger effect on both healthy and carcinoma cell lines, with the most prominent effect on the breast carcinoma MCF7 cell line. The molecular docking study, obtained for ten different conformations of the new compound, showed its inhibitory nature against CDK_S_ protein. Lower inhibition constant, relative to one of 4-OH-coumarine, proved stronger and more numerous interactions with CDK_S_ protein. These interactions were carefully examined for both parent molecule and derivative and explained from a structural point of view.

## 1. Introduction

Cell structures can be significantly influenced and damaged by sustained oxidative stress which is considered to be a major cause in the pathogenesis of many, if not all, diseases. Cancer is one of the most prominent causes of death in the modern world. Cancer initiation and progression phases have been closely related to oxidative stress which increases somatic mutations, neoplastic transformation, and generally genome instability [1, 2]. In the past several years, there have been an increased number of articles that connect oxidative stress with the development of various cancers, although the actual links are a matter of dispute [1, 2].

Nowadays, there is an increased interest in new drug discovery, to adequately promote the survival rate against cancer. Presently existing anticancer drugs are insufficient due to many side effects, nonselectivity, resistance, etc. Many of the naturally occurring molecules possessing antioxidant activity exhibit a synergistic effect when combined with other naturally occurring, or synthetic, molecules. This can lead to the creation of new types of a biological rationale for the treatment of different cancer types or their use as adjuvants with conventional therapeutic regimens. The present paper presents the design of a new coumarin/catecholamine derivative [1, 2].

Coumarins and their derivatives are widely present family of molecules in nature. They can be accumulated in fruits [3], vegetables [4, 5], trees [6], seeds [7], and vines. Coumarins have important biological activities, some of which include regulation of growth, control of respiration [8], defense against herbivores and microorganisms [9], and hormonal and signaling role [10]. The common structural elements are fused pyrone and benzene rings with a carboxyl group on the first ring. The structural diversity of coumarins allows different pharmacological properties: antibacterial, antifungal, antioxidant, and cytotoxic [11–13]. The synthetic coumarins also have great potential as drugs for the treatment of neurodegenerative [14, 15] and microbial [16, 17] diseases, HIV [18, 19], and cancer [20, 21]. They also have been used as regulators of reactive radical species [22, 23]. Several reactive derivatives of coumarin with various molecules were already synthesized, and their biological reactivity explained experimentally and theoretically [24–26].

Dopamine, on the other side, belongs to the important group of catecholamines, which function as hormones and neurotransmitters in the peripheral endocrine and central nervous system [27–30]. The other functions include control of movement, reward processing, attention, working memory, emotions, sleeping, and dreaming. Dopamine and other catecholamines, as well as their metabolites, are gaining significant attention, due to the hypothesis that the origin of some, if not all, neurodegenerative diseases lies in oxidative stress and changes at the molecular level [31–34]. More than 20% of oxygen in the body is used in the brain; therefore, a considerable amount of the present fatty acids is exposed to reactive oxygen and nitrogen species [35–38]. The low permeability of the blood-brain barrier limits the use of the external antioxidants, which increases the importance of molecules with good radical scavenging activities that are already present in the body [37]. The catechol structure, as well as various side groups, has been proven, in different experimental and theoretical studies, to enhance the antiradical potency of molecules [39–43]. Besides being good antiradical agents, catecholamines and their analogs also exhibited as potential antitumor agents [44–46]. The structure-activity studies show that catechol moiety is also the essential part of these molecules influencing their antitumor activity [45, 47, 48]. In the case of dopamine and its analogs, it has been concluded that the aminoethyl group could be replaced by methyl or aminomethyl groups without a noticeable change in activity towards P-388 leukemia cells [49]. One of the assumptions is that the formation of reactive quinone is responsible for the cytotoxic and genotoxic activity of dopamine [50].

This article presents data on the reactivity of novel coumarin/dopamine derivative towards various tumor and healthy cells lines, with special emphasis on the synergistic effect of these two molecules. The synthesis is carried out assuming that there is no formation of a quinone, which is harmful to healthy cells, and that the catechol unit is preserved and combined with the coumarin. The crystallographic structure is obtained for the synthesized derivative and explained in details. The molecular docking study is performed in order to gain better insight into the mechanism of the biological activity of new compound and possible differences in coumarin-protein and new derivative-protein interactions. The protein selected for molecular docking is cyclin-dependent kinases (CDKs), for both 4-hydroxycoumarin, as a parent molecule, and synthesized derivative. CDKs plays an important role in the control of cell division [51], and its deregulation can lead to the development of human diseases including cancer [52, 53]. Thus, the suppression of its activity could be a promising molecular route for anticancer therapy [54].

## 2. Materials and Methods

### 2.1. Substances

Dopamine hydrochloride, 4-hydroxycoumarin (4-OH-coumarin), methanol, toluene, and 96% ethanol were purchased from Sigma-Aldrich. The starting compound, 3-acetyl-4-hydroxy-coumarin, was obtained as explained in [55].

### 2.2. Spectral Analysis

The vibrational spectra were recorded on the Perkin-Elmer Spectrum One FT-IT spectrometer in the range between 4000 and 400 cm^−1^. The KBr pellet technique was used. The NMR spectra (^1^H and ^13^C) were recorded on a Varian Gemini 200 spectrometer in CDCl_3_ as a solvent and with TMS as an internal standard. The elemental microanalysis for C, H, and N was performed on the Vario EL III C, H, and N Elemental Analyzer. The mass spectra were obtained on a 5973 Mass spectrometer (Agilent, Santa Clara, CA) with MS quadruple, temperature 150°C, and a mass scan range of 40–600 amu at 70 eV.

### 2.3. Cell Culture

In this study, the following cell lines were used: healthy human keratinocyte HaCaT (AddexBio T0020001), cervical squamous cell carcinoma SiHa (ATCC®, HTB-3^5^™), breast carcinoma MCF7 (ATCC® HTB-22™), and hepatocellular carcinoma HepG2 (ATCC® HB-8065™). All cell lines were grown in Dulbecco's modified Eagle's medium (DMEM) supplemented with 10% fetal bovine serum (FBS), 4500 mg/L glucose, and 1x antimycotic/antibiotic (all from Invitrogen™, USA). The cells were maintained at 37°C in 5% CO_2_.

### 2.4. Cell Viability Assay

In order to assess whether selected compounds influence the viability of cell, the MTS Cell Proliferation Assay was applied, a colorimetric method for sensitive quantification of viable cells. The method is based on the reduction of MTS tetrazolium compound by viable cells generating a colored formazan product. This conversion is thought to be carried out by NAD(P)H-dependent dehydrogenase enzymes in metabolically active cells. The formazan dye produced by viable cells can be quantified by measuring the absorbance at 490–500 nm.

Cells (5 × 10^3^ for Siha and MCF7 and 1 × 10^4^ for HaCaT and HepG2/per well) were seeded in 96-well plates a day before treatment and then treated with various concentrations of 4-OH-coumarin or derivative **3** (100, 300, and 500 *μ*M) for 48 hours. After 48 h, the effect of these treatments was monitored on cell's viability using MTS Cell Proliferation Assay (Promega CellTiter 96® AQueous One Solution Cell Proliferation Assay) and colorimetric quantification was done using a plate reader (Plate Reader Infinite 200 pro, Tecan).

### 2.5. Statistical Analyses

Statistical analyses were performed with SPSS statistical software (version 20). The data represent the means ± SEM from at least three independent experiments. Statistical analyses were performed by Student's *t*-test, and *p* value ≤ 0.05 was considered significant.

### 2.6. Theoretical Methods

Software package AutoDock 4.0 was used for molecular docking simulations [56]. The three-dimensional crystal structure of CDK protein was procured from the Protein Data Bank (PDB ID: 1KE9). Preparation of protein for docking simulations was done by removing the cocrystallized ligand, water molecules, and cofactors in the Discovery Studio 4.0. The calculations of Kollman charges and adding of the polar hydrogen were performed using the AutoDockTools (ADT) graphical user interface. The optimization of coumarin and coumarin derivate structures was performed at B3LYP-D3BJ/6-311++G(d,p) level of the theory [57–61] in the gas phase. For this purpose, Gaussian 09 Program package [62] was used. The structure of coumarin derivate was taken from crystallographic data. This level of theory was proven to reproduce well the crystallographic structure and experimentally obtained spectra for similar compounds [24, 25, 26]. The docking simulations were completed independently. During these simulations, the protein retained a rigid structure in the ADT, while both docked compounds possessed flexibility. All bonds of the compounds were treated as rotatable. For calculations of partial charges, the Geistenger method was selected. The Lamarckian Genetic Algorithm (LGA) method was used in all calculations for protein-ligand flexible docking simulation [63]. The grid boxes with grid center 4.0 × 50.3 × 11.5 of CDK protein covered all of the protein binding sites and enabled free motion of ligands. The interactions between CDKs and the corresponding ligands were estimated and analyzed, for several most stable conformations, through the positions of the amino acids and the type of interaction that is achieved.

### 2.7. General Procedure for the Synthesis of 3-(1-((3,4-Dihydroxyphenethyl)amino)ethylidene)-chroman-2,4-dione

**Scheme 1:**
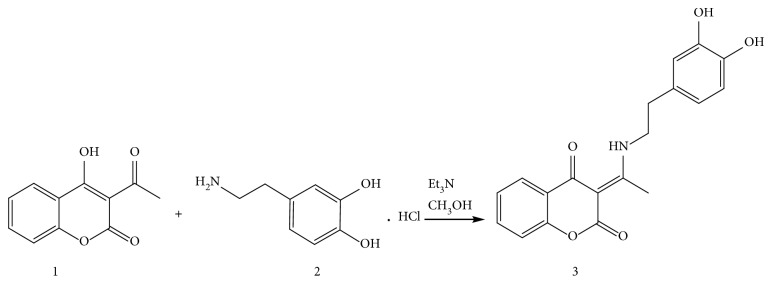
Synthesis of 3-(1-((3,4-dihydroxyphenethyl)amino)ethylidene)-chroman-2,4-dione.

The synthesis of new coumarin derivative is presented in Scheme 1. The new compound (designated as compound **3** in Scheme 1) was obtained by refluxing and mixing of the reaction mixture (3-acetyl-4-hydroxicumarine (0.0014 mol; 0.3 g), dopamine hydrochloride (0.0014 mol; 0.23 g), and equimolar amounts of triethyl amine (0.0014 mol; 0.15 g) in 50 ml methanol for 3 h. The progress of reactions was monitored by TLC (toluene: acetone = 7: 3). When the reaction was completed, the mixture was cooled to room temperature. The obtained white crystals were filtered, air-dried, and recrystallized from methanol.

Yield, 0.321 g (64.45 %), Anal. Calcd. for C_19_H_17_O_5_N (*M*_r_ = 339.32) %: C, 67.25; H, 5.05; N, 4.12. Found: C, 67.00; H, 5.10; N, 4.16.

^1^H NMR (DMSO, 200 MHz) *δ* in ppm: 2.55 (s, 3H, C2′–H), 2.80 (t, 2H, C2^″^–H), 3.76 (q, 2H, C1^″^–H), 6.5 (m, 1H, C4^″^–H, J=2.0 Hz), 6.67 (m, 2H, C7^″^–H, C8^″^–H), 7.25 (m, 1H, C6–H), 7.60 (dd, 2H, C5–H, C7–H), 7.91 (m, 1H, C8–H), 8.81 (s, 2H, OH), 13.66 (s, 1H, NH)

^13^C NMR (DMSO, 50 MHz) *δ* in ppm: 18.35 (C2′), 34.11 (C2^″^), 45.71 (C1^″^), 96.18 (C3), 115.87 (C7^″^), 116.41 (C8), 119.74 (C8^″^), 120.41 (C10), 123.78 (C6), 125.86 (C5), 128.12 (C3^″^), 134.12 (C7), 144.17 (C6^″^), 145.46 (C5^″^), 153.20 (C9), 162.13 (C2), 1776.28 (C1′), 179.60 (C4).

IR (KBr, cm^−1^): 3305 (NH and OH), 2921, 2857 (CH), 1668 (C=O), 1605, 1569, 1531, (C=C), 1118 (C–O).

### 2.8. X-ray Data Collection and Structure Refinement

A summary of X-ray diffraction experiment and structure refinement for **3·MeOH** is given in Table 1. The data collection was performed on an Oxford Diffraction Xcalibur Gemini ultra-diffractometer equipped with an AtlasS2 CCD detector using CuK*α* radiation. CrysAlis PRO 1.171.39.35c [64] was used for data collection, cell refinement, data reduction, and absorption correction. The structure was solved by SHELXT [65] and subsequent Fourier syntheses using SHELXL [66], implemented in WinGX program suit [67]. Anisotropic displacement parameters were refined for all nonhydrogen atoms. The hydrogen atoms bonded to nitrogen, oxygen atoms were found in the Fourier maps and refined freely, and aromatic and aliphatic carbon-bonded hydrogen atoms were placed in the calculated positions and refined riding on their parent C atoms with corresponding C–H distances and *U*iso(H) = 1.2 or 1.5 *U*eq(C), respectively. The analysis of bond distances and angles was performed using SHELXL and PLATON [68]. DIAMOND [69] was used for molecular graphics.

## 3. Results and Discussion

### 3.1. Chemistry

The synthesis of coumarin derivative is presented in Scheme 1. The structure of synthesized compound **3** was determined by means of elemental, spectral (IR, ^1^H NMR, and ^13^C NMR), and X-ray structural analysis (in the form of **3·MeOH**).

The formation of compound **3** was confirmed by IR spectra, with the presence of bands positioned at 3305 cm^−1^ assigned to NH and OH group vibrations. Also, stretching vibrations corresponding to the C=O and C–O groups from 2,4-dioxochroman moiety were identified at 1668 and 1118 cm^−1^, respectively.

In ^1^H NMR spectrum, the singlet positioned at 2.55 ppm was assigned to protons on C2′ atom. The resulting signals of aromatic protons of the 2,4-dioxochroman part were in the range from 7.25 to 7.91 ppm. Aromatic protons belonging to the phenyl group of dopamine part were detected in the range from 6.5 to 6.67 ppm. The protons of the methylene C2^″^–H and C1^″^–H groups were identified as triplets at 2.80 and 3.6 ppm. Signals of protons of the phenyl OH and enamine NH group were identified as broadened singlets at 8.81 and 13.66 ppm.

The ^13^C NMR spectra of compound **3** indicated the presence of aromatic carbon atoms of the dopamine part in the range from 115.87 to 145.46 ppm. The signals of carbon atoms of coumarine moiety were identified in the range from 96.18 to 179.60 ppm. The carbons of lactone (C–2) and ketone (C–4) showed resonances at 162.13 and 179.60 ppm. Signals at 18.35, 34.11, and 45.71 ppm were assigned to C2′, C1^″^, and C2^″^ carbons, respectively.

### 3.2. Crystallographic Structure

**Figure 1:**
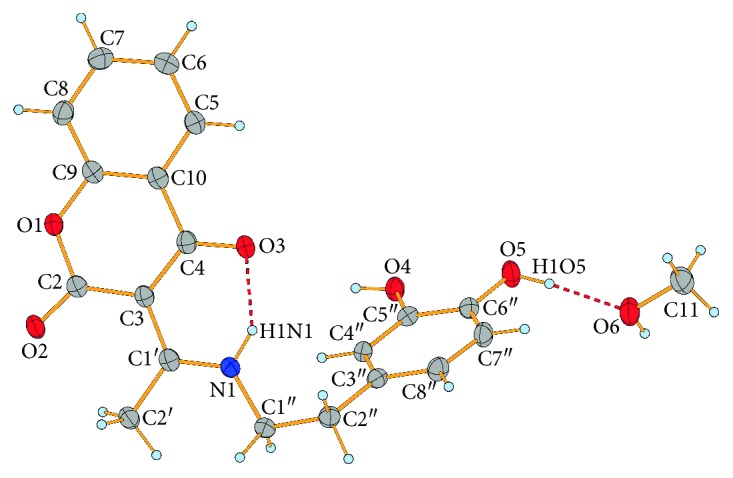
Molecular structure with an atom numbering scheme of **3·MeOH**. Displacement ellipsoids are drawn at 50% probability; hydrogen bonds are shown as red dashed lines.

X-ray structure analysis revealed that **3** crystallizes in the triclinic *P*-1 space group. Its molecular structure is formed by the molecule of **3**, consisting of bicyclic coumarine and 3,4-dihydroxyphenethyl fragments joined by aminoethylidine chain, and by a solvated molecule of methanol tied with the mentioned molecule by a hydrogen bond (Figure 1). Within the molecule, the dihedral angle between the planes of coumarin fragment and the 3,4-dihydroxyphenyl ring is 61.46(3)°.

A typical structural feature of that type of compounds is an intramolecular N − H···O hydrogen bond which forms a six-membered ring with S(6) graph-set motif [70] and thus the molecule occurs in a ketoamine tautomeric form. Considering the O3=C4−C3=C1′−N1−H1N1-conjugated bond ring system created, owing to the intramolecular hydrogen bond formation, the equalization of the C3−C4 and C3=C1′ (both 1.442(2) Å) bond lengths (Table S1) is observed although the bonds are formally single and double, respectively. This can be explained by the *π*-electron delocalization within the system, and thus, we may conclude that the above hydrogen bond can be classified as a resonance-assisted hydrogen bond [71]. Interestingly, in the related 3-(1-((m-toluidine)amino)ethylidene)-chroman-2,4-dione and in 3-(1-(2-hydroxyethylamino)ethylidene)-chroman-2,4-dione [72] compounds, C3−C4 bond (1.434(2) and 1.430(3) Å, respectively) is even slightly shorter than C3=C1′ bond (1.436(2) and 1.437(3) Å, respectively). The elongation of C4=O3 (1.248(2) Å) bond, which is markedly longer than C2=O2 (1.223(2) Å) bond not involved in a strong hydrogen bond, and shortening of C1′−N1 (1.309(2) Å) bond in **3** can also be observed. Very similar bond lengths were observed in related compounds, like 3-(1-((o-toluidine)amino)ethylidene)-chroman-2,4-dione [72], 3-(1-(phenylamino)ethylidene)-chroman-2,4-dione [24], 3-(1-(3-hydroxypropylamino)propylidene)chroman-2,4-dione [73], 2-(1-(2,4-dioxochroman-3-ylidene)ethylamino)-3-methylbutanoate [74], 3-[(1-benzylamino)ethylidene]-2*H*-chromene-2,4(3*H*)-dione [75], or 3-[1-((2-hydroxyphenyl)amino)ethylidene]-2*H*-chromene-2,4(3*H*)-dione compound [76]. All other bond lengths and angles (Table S1) in the molecule of **3** are within normal ranges [77].

**Figure 2:**
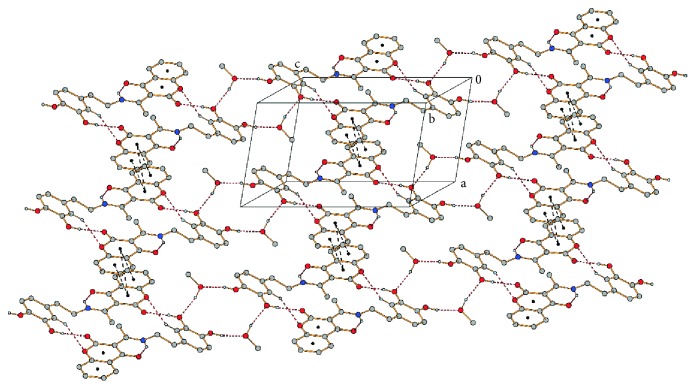
Molecular packing of **3·MeOH** showing *π*-*π* interactions (black dashed lines) between neighboring chains formed by hydrogen bonds (red dashed lines). Hydrogen atoms not involved in hydrogen bonds are omitted for clarity.

Except for above discussed N1−H1N1···O3 intramolecular hydrogen bond, due to which the exocyclic C3=C1′ double bond has an *E* geometry, the molecules of **3** are stabilized in the solid state by intermolecular O−H···O and C−H···O hydrogen bonds. Further stabilization of the solid state structure occurs when molecule of methanol occupies empty space between molecules of **3** and is tied with the molecules by a pair of O−H···O hydrogen bonds (Table 2). Due to these bonds, the molecules of **3** and methanol are tied to form chains parallel with the [011] direction (Figure S2). These chains are further connected by *π*-π interactions between pyran-2,4-dione (py) and phenyl (ph) rings of coumarin moieties in adjacent chains into a 2D structure parallel with (011) (Figure 2). These *π*-*π* interactions are characterized by Cg_py_···Cg_py_^iii^ and Cg_py_···Cg_ph_^iii^ centroid-centroid distances of 3.6479(1) and 3.7569(1) Å, respectively (iii = 1–*x*, 1–*y*, 1–*z*).

### 3.3. Antitumor Activity

**Figure 3:**
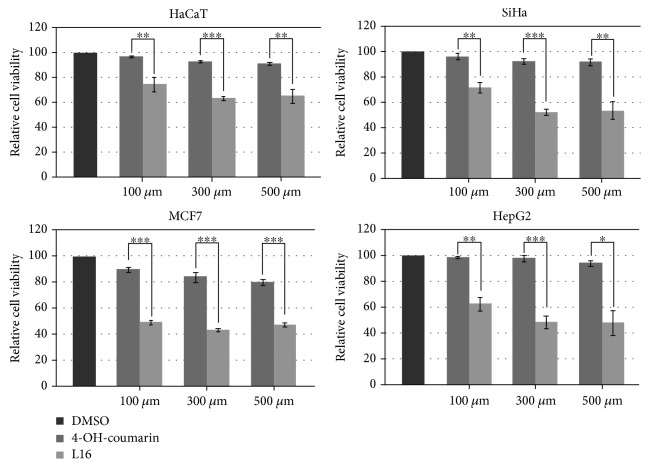
Cell viability assay (MTS assay) on HaCaT, SiHa, MCF7, and HepG2 cells performed 48 h after treatment with either 4-OH-coumarin or compound **3**. Relative cell viability of cells treated with selected compounds was calculated as a percentage of DMSO-treated cell viability that was set as 100%. Data are presented as the means ± S.E.M. (standard error mean) of at least three independent experiments performed in triplicate for each concentration. Mean values of relative cell viability were compared with Student's *t*-test, and *p* values are presented as ^∗^ *p* ≤ 0.05, ^∗∗^ *p* ≤ 0.01, and ^∗∗∗^ *p* ≤ 0.001. Each color corresponds to a bar presented on the histogram.)

The effect of both 4-OH coumarin and its derivate **3** on selected cell lines was monitored and compared to the effect of DMSO that was used as a control (vehicle). As presented in Figure 3, 4-OH-coumarin exhibited a mild effect on cell's viability in all tested cell types with the maximal effect on breast carcinoma cell line MCF7, inducing 20% reduction in viability of these cells 48 h after treatment at 500 *μ*M concentration (*p* = 0,023). On the other hand, its derivate **3** had, in comparison with the control sample, a significantly stronger effect on both healthy and carcinoma cell lines with the most prominent effect on the MCF7 cell line. In particular, 100 *μ*M concentration of derivative **3** led to the reduction of cell's viability to approximately 75% in HaCat cells (*p* = 0,023), 72% in SiHa cells (*p* = 0,008), 49% in MCF7 cells (*p* = 0,008), and 62% in HepG2 cells (*p* = 0,005). The reduction effect increased with the concentration of the derivative **3** and had the greatest effect on the MCF7 cells, with a reduction percentage of 43% at the concentration of 300 *μ*M (*p* = 0.001). The statistical significance is also observed when the effect of **3** is compared with the effect of 4-OH–coumarin (Figure 3, *p* values are indicated as asterisks). Taken together, the higher cytotoxic effect of derivate **3** is observed, both for healthy and for carcinoma cells, compared to 4-OH-coumarin, where the effect on the carcinoma cells was somewhat stronger.

It is clearly demonstrated that coumarin derivate **3** exhibits a cytotoxic activity against all analyzed cell types *in vitro*. The observed effect is somewhat stronger against carcinoma cell lines compared to healthy keratinocytes, with the most prominent effect on breast carcinoma cell line MCF7. Antitumor activity of natural and synthetic coumarin derivatives has been previously reported by various authors including high cytotoxicity against ovarian cancer cells, lung carcinoma cells, pancreatic carcinoma, hepatocarcinoma, and breast and colon carcinoma [78–82]. Depending on their structures, coumarins can act on various tumor cells by different mechanisms such as inhibition of the telomerase and protein kinase activities, downregulation of oncogene expression or induction of the caspase-9-mediated apoptosis, and suppression of cancer cell proliferation [82]. A further work is needed to elucidate the mechanism of action for this newly synthesized derivate **3**.

### 3.4. Molecular Docking

**Figure 4:**
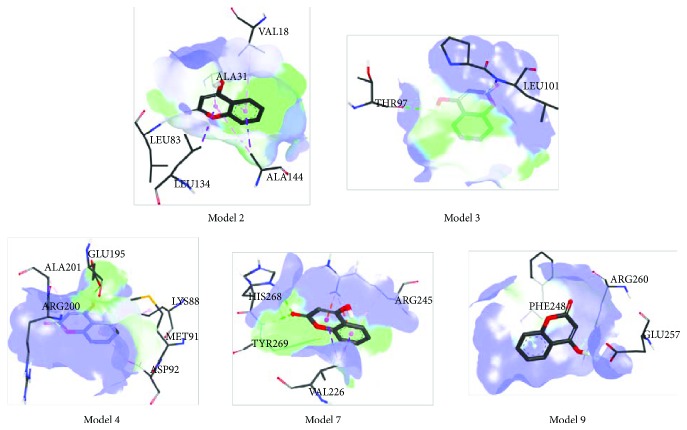
Docking positions of 4-OH-coumarin.

**Figure 5:**
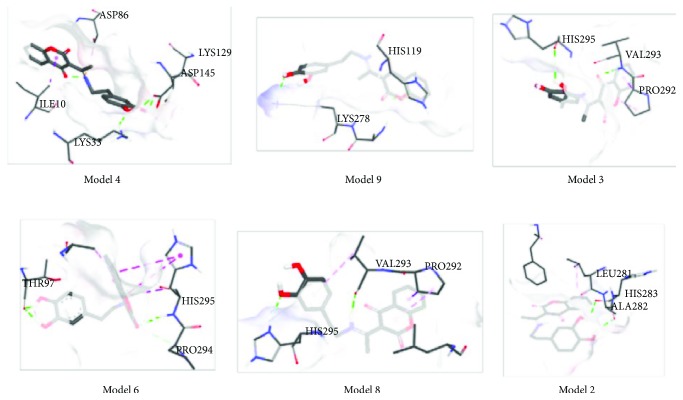
Docking positions of coumarin derivate.

The molecular docking studies were performed for the evaluation of the inhibitory nature of examined compounds against CDK_S_ protein. These simulations gave the predicted protein-ligand binding energies and identified the potential ligand binding sites. The structure of the newly synthesized compound was optimized at the B3LYP-D3BJ/6-311++G(d,p) level of theory, based on the crystallographic positions of atoms (Tables S3 and S4). Ten different conformations were analyzed for both investigated compounds. Tables S5 and S6 give values of the estimated free energy of binding and inhibition constant values (*K*_i_), the distance between respective active sites of ligand and amino acids, and pairwise interaction energies (*E*_i_), as well as types of interactions for the investigated models with the lowest docked conformation energies. The most stable conformations are presented in Figures 4 and 5.

The parent compound contains several polar groups, namely, a hydroxy group in position 4, a carbonyl group, and an oxygen atom in the pyrone ring. Since the rest of the structure makes the benzene ring, therefore, the most of interactions, presented in Table S5, include *π*-alkyl and *π*-*σ* hydrophobic interactions with leucine, valine, alanine, arginine, and phenylalanine in various positions [83–85]. These interactions are characterized by low pairwise interaction energy and large atomic distances (≥2.5 Å). Due to a large number of weak noncovalent interactions, all of the models obtained for 4-OH-coumarin have very low binding energy and high value of inhibition constant, which is in accordance with the obtained experimental result.

It is also evident that two types of hydrogen bonds are formed in given models. The first type is the conventional hydrogen bond. Only two bonds of this type, with significant values of pairwise interaction energy, have atomic distances lower than 2 Å. These bonds are formed upon the interaction between CDKs and LEU83 and GLU257. It should be pointed out that in models 3 and 4 there are two more bonds which deserve attention, THR97 and GLU195, respectively. It should be pointed out that the hydroxy group of 4-OH-coumarine behaves as the hydrogen atom donor in the interaction with THR97 (model 2), GLU195 (model 3), and GLU257 (model 9). There are more interactions in which the parent molecule behaves as the hydrogen atom acceptor. Other conventional hydrogen bonds are weak, due to high atomic distances and low pairwise interaction energy. These interactions are established with positively charged amino acids, lysine, histidine, and arginine. The second type of hydrogen bonds is the carbon-hydrogen bond. Predicted values of the interaction energy for this bond type are very low, and the atomic distances much larger (≥3 Å). These bonds are formed between donating groups of HIS268 and ARG260 and hydrogen-acceptor groups of the CDKs.

Table S6 presents molecular docking results for compound **3**. When the structures of two investigated compounds are compared, it is notable that only compound **3** possesses a catechol moiety and longer aliphatic chain with a nitrogen atom. This increases the number of possible interactions with amino acids. If results from Tables S5 and S6 are compared, it is obvious that the relative abundance of hydrogen bonds increases with respect to other noncovalent interactions. There are also still hydrophobic *π*-*σ* (ILE10 in model 4 and PRO292 in model 8) and hydrophobic *π*→alkyl (in models 2, 3, 8, and 9) interactions. All these interactions have very low pairwise interaction energies. When these interactions are formed, atomic distances fall into the very wide range of values (3.5-4.40 Å). In spite the fact that these interactions are very weak, they additionally stabilize the structures.

When hydrogen bonds are concerned, there are also two types of bonds, the conventional and carbon-hydrogen bonds. Compound **3** behaves as the hydrogen atom donor with ASP in positions 145 and 86 (model 4), THR97 (model 6), VAL293 (model 8), ALA282, and HIS293 (model 2). It is important to point out that the number of possible hydrogen atom accepting amino acids increases, due to the fact that compound **3** has additional polar groups. The strength of formed conventional hydrogen bonds and interatomic distances are in the same range as in 4-OH-coumarine. Therefore, it can be concluded that the reactivity of coumarin part is conserved and that the additional groups contribute to the increasing number of interactions.

It should be emphasized that both values, the binding energy and inhibition constant, of the compound **3** are lower in comparison with the parent molecule. Moreover, it should be pointed out that the value of inhibition constant for compound **3** is more than six times lower than the one of 4-OH-coumarine. This indicates that compound **3** interacts better with CDK_S_ protein. Obviously, the additional hydroxy groups and an aromatic ring, with an aliphatic chain containing a nitrogen atom, allow new interactions which inhibit the reactivity of the protein. This could be one of the reasons for the increased reactivity of compound **3** towards tumor cells.

## 4. Conclusion

The coumarin and dopamine derivative, 3-(1-((3,4-dihydroxyphenethyl)amino)-ethylidene)-chroman-2,4-dione, was synthesized under mild conditions. The new compound was analyzed by NMR, IR, microanalysis, and X-ray crystallography. The X-ray analysis showed that the similar structural motifs are present, as with other previously obtained derivatives with aminophenols. Several types of hydrogen bonds, both intramolecular and intermolecular, stabilize the structure within the crystal. The molecule is not planar, but there is the dihedral angle between the planes of coumarin fragment and 3,4-dihydroxyphenyl ring of 61.46(3)°. There are also *π*→*π* and stacking interactions within the crystal structure.

The antitumor activity was investigated against healthy and tumor cell lines both for 4-OH-coumarin and its derivative. Compared to 4-OH coumarin, new derivative showed a significantly stronger effect on both healthy and carcinoma cell lines. When treated with 100 *μ*M solution of compound **3**, the reduction of cell's viability was approximately 75% in HaCat cells, 72% in SiHa cells, 49% in MCF7 cells, and 62% in HepG2. The most prominent effect was observed on the breast carcinoma MCF7 cell line.

The molecular docking study was performed in order to better understand the difference in binding between the two investigated molecules and CDK protein. Different interactions are possible due to the presence of polar groups in the coumarin structure. The hydrogen bonds are the strongest interactions observed, in which 4-OH-coumarin can act as the hydrogen atom acceptor and hydrogen atom donor. The most numerous are *π*→alkyl interactions with various amino acids. The strength of interactions, given as the pairwise interaction energy, is preserved in coumarin derivative. New interactions are established as a result of the presence of additional polar groups: catechol moiety and alkyl chain. The obtained result implies that a number of interactions determine the activity towards investigated protein.

In the end, it can be concluded that the presented results are promising. Future experiments, which would include new cell lines and new coumarin-neurotransmitter derivatives, in addition to all the above levels of testing, will be also supplemented with classical MD calculations in order to better explain the mechanisms of action.

## Figures and Tables

**Table 1:** Crystal data and structure refinement of **3·MeOH**.

Compound	**3·MeOH**	
Empirical formula	C_20_H_21_NO_6_	
Formula weight	371.38	
Temperature	120(2) K	
Wavelength	1.54184 Å	
Crystal system	Triclinic	
Space group	*P*-1	
Unit cell dimensions	*a* = 7.9449(3) Å	*α* = 82.538(3)°
	*b* = 8.8742(3) Å	*β* = 82.296(3)°
	*c* = 13.1920(5) Å	*γ* = 70.039(3)°
Volume	862.79(6) Å^3^	
*Z*; density (calculated)	2; 1.430 g·cm^−3^	
Absorption coefficient	0.883 mm^−1^	
*F*(000)	392	
Crystal shape, color	Prism, white	
Crystal size	0.311 × 0.201 × 0.072 mm^3^	
*θ* range for data collection	3.395–67.361°	
Index ranges	-9 ≤ *h* ≤ 6, -10 ≤ *k* ≤ 10, -15 ≤ *l* ≤ 15	
Reflections collected/independent	8977/3086 [*R*(int) = 0.0226]	
Absorption correction	Analytical	
Max. and min. transmission	0.938 and 0.841	
Data/restraints/parameters	3086/0/262	
Goodness-of-fit on *F* ^2^	1.034	
Final *R* indices [*I* > 2*σ*(*I*)]	*R*1 = 0.0342, w*R*2 = 0.0876	
*R* indices (all data)	*R*1 = 0.0398, w*R*2 = 0.0924	
Largest diff. peak and hole	0.226; -0.255 *e*.Å^−3^	

**Table 2:** Hydrogen bonds for **3·MeOH** [Å and °].

D−H···A	*d*(D−H)	*d*(H···A)	*d*(D···A)	<(DHA)
N1−H1N1···O3	0.91 (2)	1.76 (2)	2.550 (1)	143.7 (16)
O5−H1O5···O6	0.87 (2)	1.80 (2)	2.661 (1)	169.2 (18)
O4−H1O4···O2^i^	0.84 (2)	1.88 (2)	2.700 (1)	167.5 (18)
O6−H1O6···O4^ii^	0.88 (2)	1.92 (2)	2.786 (1)	167.0 (19)
C4^″^−H4^″^···O1^i^	0.95	2.57	3.417 (2)	148.6

Symmetry transformations used to generate equivalent atoms: −*x* + 2, −*y +* 1, −*z* + 1; (ii): −*x* + 2, −*y* + 2, −*z* + 2.

## Data Availability

The X-ray structure analysis and molecular docking data used to support the findings of this study are included within the supplementary information file.

## Supplementary Materials

Supplementary Materials The Supplementary information file contains additional information on crystallographic data of the newly synthesized molecule, along with selected bond lengths and angles, as well as results of docking analysis with CDKs (type of interaction, atom distances, bonding energy, estimated free energy of binding, and inhibition constants).
