# Supplementary material for: Synthesis and Characterization of 3-(1-((3,4-Dihydroxyphenethyl)amino)ethylidene)-chroman-2,4-dione as a Potential Antitumor Agent
Source: Oxid Med Cell Longev. 2019 Feb 13;2019:2069250. doi: 10.1155/2019/2069250 (PMC6393868; doi:10.1155/2019/2069250)
Supplement: Supplementary Materials — The Supplementary information file contains additional information on crystallographic data of the newly synthesized molecule, along with selected bond lengths and angles, as well as results of docking analysis with CDKs (type of interaction, atom distances, bonding energy, estimated free energy of binding, and inhibition constants). [file 2069250.f1.pdf]

Supplementary information for
Synthesis and characterization of 3-(1-((3,4-dihydroxyphenethyl)amino)ethylidene)-chroman-2,4-dione as potential anti-tumor agent

Dušan S. Dimić[1], Zoran S. Marković[2], Luciano Saso[3], Edina H. Avdović[4], Jelena R. Đorović[5], Isidora P. Petrović[6], Danijela D. Stanisavljević[6], Milena J. Stevanović[6-8], Ivan Potočňák[9], Erika Samoľová[9], Srećko R. Trifunović[4], Jasmina M. Dimitrić Marković[1*]

[1]*University of Belgrade, Faculty of Physical Chemistry, Studentski trg 12-16, 11000 Belgrade, Serbia*

[2]*Department of Chemical-Technological Sciences, State University of Novi Pazar, Vuka Karadžića bb, 36300 Novi Pazar, Serbia*

[3]*Department of Physiology and Pharmacology "Vittorio Erspamer", Sapienza University of Rome, Rome, Italy*

[4]*University of Kragujevac, Faculty of Science, Radoja Domanovića 12, 34000 Kragujevac, Serbia*

[5]*BioIRC, Bioengineering R&D Center, Prvoslava Stojanovića 6, 34000 Kragujevac, Serbia*

[6]*University of Belgrade, Institute of Molecular Genetics and Genetic Engineering, Vojvode Stepe 444a, PO Box 23, 11010 Belgrade, Serbia*

[7]*University of Belgrade, Faculty of Biology, Studentski trg 16, 11000 Belgrade, Serbia*

[8]*Serbian Academy of Sciences and Art, Knez Mihajlova 35, 11000 Belgrade, Serbia*

[9]*Institute of Chemistry, P. J. Šafárik University in Košice, Moyzesova 11, 04154 Košice, Slovak Republic*

CCDC 1855089 contains the supplementary crystallographic data for **3**. These data can be obtained free of charge via http://www.ccdc.cam.ac.uk/conts/retrieving.html, or from the Cambridge Crystallographic Data Centre, 12 Union Road, Cambridge CB2 1EZ, UK; fax: (+44) 1223-336-033; or e-mail: deposit@ccdc.cam.ac.uk.

Table S1. Selected bond lengths [Å] and angles [°] for **3·MeOH**.

O3−C4	1.2483(15)	O3−C4−C3	123.95(12)
C4−C10	1.4730(18)	O3−C4−C10	118.68(11)
C4−C3	1.4420(18)	C3−C4−C10	117.37(11)
C3−C1'	1.4418(18)	C1'−C3−C4	120.27(11)
N1−C1'	1.3094(17)	N1−C1'−C3	117.93(11)
N1−C1"	1.4743(16)	C1'−N1−C1"	127.09(11)
C1'−C2'	1.4988(18)	N1−C1'−C2'	118.88(11)
C3−C2	1.4389(17)	C1'−C3−C2	119.54(11)
O1−C2	1.3820(16)	C4−C3−C2	120.19(11)
O1−C9	1.3756(15)	C3−C1'−C2'	123.19(11)
O2−C2	1.2225(15)	O2−C2−C3	127.84(12)
C9−C10	1.3852(18)	O1−C2−C3	118.87(11)
O4−C5"	1.3762(15)	O2−C2−O1	113.29(11)
O5−C6"	1.3669(16)	C9−O1−C2	122.55(10)
		O1−C9−C10	121.52(11)
		C9−C10−C4	119.49(11)

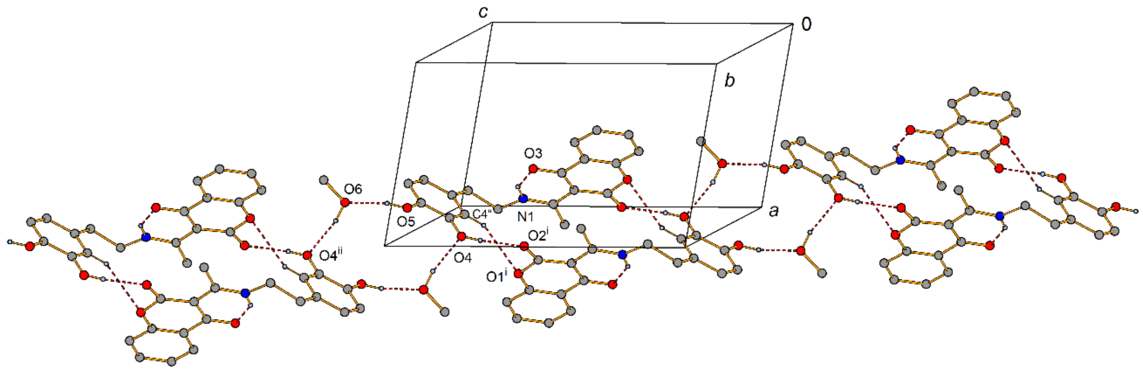

Figure S2. A chain parallel with the [011] direction in the crystal structure of **3·MeOH** showing hydrogen bonds (red dashed lines). Hydrogen atoms not involved in hydrogen bonds are omitted for clarity. Symmetry codes: (i): -*x* + 2, -*y* + 1, -*z* + 1; (ii): -*x* + 2, -*y* + 2, -*z* + 2.

Table S3. Atomic coordinates (x 10^4) and equivalent isotropic displacement parameters (Å^2 x 10^3) for **3·MeOH**. *U*(eq) is defined as one third of the trace of the orthogonalized U^{ij} tensor.

Atom	*x*	*y*	*z*	*U*(eq)
O1	7543(1)	4443(1)	3584(1)	23(1)
O4	9726(1)	8787(1)	7774(1)	22(1)
O2	9530(1)	2212(1)	4134(1)	26(1)
O3	6556(1)	6354(1)	6339(1)	27(1)
O5	7865(1)	10199(1)	9411(1)	27(1)
O6	7455(1)	10809(1)	11368(1)	31(1)
N1	8765(2)	3760(1)	7100(1)	21(1)
C9	6321(2)	5958(2)	3693(1)	20(1)
C4	6904(2)	5727(2)	5509(1)	20(1)
C5"	8908(2)	7906(2)	8479(1)	19(1)
C10	5955(2)	6637(2)	4620(1)	20(1)
C4"	9064(2)	6330(2)	8364(1)	19(1)
C6"	7930(2)	8657(2)	9340(1)	21(1)
C2	8499(2)	3518(2)	4377(1)	20(1)
C3	8200(2)	4157(2)	5358(1)	19(1)
C1'	9178(2)	3201(2)	6195(1)	19(1)
C2'	10642(2)	1617(2)	6076(1)	23(1)
C8"	7213(2)	6240(2)	9934(1)	24(1)
C3"	8210(2)	5474(2)	9087(1)	20(1)
C1"	9515(2)	2952(2)	8068(1)	23(1)
C5	4698(2)	8187(2)	4678(1)	22(1)

C2"	8338(2)	3751(2)	8984(1)	23(1)
C6	3860(2)	9018(2)	3822(1)	26(1)
C7	4250(2)	8307(2)	2899(1)	27(1)
C8	5480(2)	6773(2)	2829(1)	25(1)
C7"	7072(2)	7808(2)	10064(1)	25(1)
C11	6009(2)	11858(2)	11960(1)	35(1)

Table S4. The DFT Cartesian coordinates of the optimized structure of **3**

Atom	*x*	*y*	*z*
O1	-3.578	0.782	1.338
O4	5.574	-0.544	1.563
O2	-2.345	2.491	1.961
O3	-0.962	-0.336	-1.607
O5	4.164	-2.793	1.691
N1	0.512	1.637	-1.021
C9	-3.831	-0.316	0.574
C4	-1.738	0.042	-0.700
C5"	4.524	-0.675	0.700
C10	-2.961	-0.728	-0.435
C4"	4.170	0.301	-0.224
C6"	3.780	-1.865	0.767
C2	-2.442	1.523	1.180
C3	-1.510	1.215	0.119
C1'	-0.355	2.029	-0.090

C2'	-0.088	3.284	0.688
C8"	2.363	-1.058	-1.028
C3"	3.089	0.128	-1.098
C1"	1.785	2.284	-1.373
C5	-3.820	-1.872	-1.182
C2"	2.695	1.241	-2.040
C6	-4.441	-2.580	-0.920
C7	-5.302	-2.149	0.098
C8	-5.004	-1.020	0.848
C7"	2.709	-2.041	-0.101
C11			

Table S5. Results of docking analysis of the investigated coumarine with CDKs: model with its role in bond formation; corresponding amino acid with its position in the structure of protein, and with its role in bond formation; type of the interaction during bond formation; distance in Å between respective active cites of ligand and amino acids; pairwise interaction energy (E_i) in kJ/mol; estimated free energy of binding (E_b) in kJ/mol, estimated inhibition constant (K_i) in µM.

**coumarine**		**Amino acid**		**Type of interaction**	**Atom distance (Å)**	**E_i (kJ/mol)**	**E_b (kJ/mol)**	**K_i (µM)**
Model 2							-17.87	746.49
	H-Acceptor	LEU83	H-Donor	Conventional Hydrogen Bond	1.83	2.93		
	π –Orbitals are electron acceptors	LEU134	C-H bond is electron donor	Hydrophobic π - σ	3.65	-0.08		
	π –Orbitals are electron acceptors	ALA144	C-H bond is electron donor	Hydrophobic π - σ	3.89	-0.08		
	π –Orbitals are electron donors		Alkyl group is electron acceptor	Hydrophobic π -Alkyl	4.96	-0.04		
	π –Orbitals are electron donors	VAL18	Alkyl group is electron acceptor	Hydrophobic π -Alkyl	4.84	-0.08		
	π –Orbitals are electron donors	ALA31	Alkyl group is electron acceptor	Hydrophobic π -Alkyl	3.74	-0.08		
	π –Orbitals are electron donors		Alkyl group is electron acceptor	Hydrophobic π -Alkyl	4.60	-0.04		
Model 3							-17.41	890.62
	H-Donor	THR97	H-Acceptor	Conventional Hydrogen Bond	2.55	1.80		
	π –Orbitals are electron acceptors	LEU101	H-Donor	π -Donor Hydrogen Bond	2.49	3.05		
Model 4							-16.69	1.18×10^{3}
	H-Donor	GLU195	H-Acceptor	Conventional Hydrogen Bond	2.43	3.26		
	H-Acceptor	LYS88	H-Donor	Conventional Hydrogen Bond	3.36	-0.13		
	H-Acceptor	ARG200	H-Donor	Conventional Hydrogen Bond	3.28	-0.08		
	π –Orbitals are electron	ASP92	Negative	Electrostatic π -	4.22	-0.13		

	acceptors			Anion				
	π –Orbitals are electron donors	ALA201	Alkyl group is electron acceptor	Hydrophobic π -Alkyl	4.47	-0.08		
	π –Orbitals are electron donors	MET91	Alkyl group is electron acceptor	Hydrophobic π -Alkyl	4.72	-0.08		
Model 7							-16.07	1.54 x10^3
	H-Acceptor	HIS268	H-Donor	Conventional Hydrogen Bond	2.30	0.08		
	H-Acceptor		H-Donor	Carbon Hydrogen Bond	3.62	-0.04		
	H-Acceptor	TYR269	H-Donor	Conventional Hydrogen Bond	2.28	0.08		
	π –Orbitals are electron acceptors	ARG245	Positive	Electrostatic π -Cation	3.40	-0.08		
	π –Orbitals are electron acceptors		Positive	Electrostatic π -Cation	3.85	-0.13		
	π –Orbitals are electron acceptors	VAL226	C-H bond is electron donor	Hydrophobic π - σ	3.62	-0.04		
	π –Orbitals are electron acceptors		C-H bond is electron donor	Hydrophobic π - σ	3.81	-0.13		
Model 9							-15.65	1.80 x10^3
	H-Acceptor	ARG260	H-Donor	Conventional Hydrogen Bond	2.33	0.08		
	H-Acceptor		H-Donor	Carbon Hydrogen Bond	3.51	-0.13		
	H-Donor	GLU257	H-Acceptor	Conventional Hydrogen Bond	1.95	1.51		
	π –Orbitals are electron acceptors	PHE248	H-Donor	π -Donor Hydrogen Bond	2.66	1.00		

Table S6. Results of docking analysis of the investigated coumarine derivate with CDKs: model with its role in bond formation; corresponding amino acid with its position in the structure of protein, and with its role in bond formation; type of interaction during bond formation; distance in Å between respective active cites of ligand and amino acids; pairwise interaction energy (E_i) in kJ/mol; estimated free energy of binding (E_b) in kJ/mol, estimated inhibition constant (K_i) in µM.

**Compound 3**		**Amino acid**		**Type of interaction**	**Atom distance (Å)**	**E_i (kJ/mol)**	**E_b (kJ/mol)**	**K_i (µM)**
Model 4							-22.30	122.97
	H- Donor, Positive	ASP86	H- Acceptor, Negative	Hydrogen Bond;Electrostatic Salt Bridge;Attractive Charge	2.37	3.26		
	H- Acceptor	LYS33	H- Donor	Conventional Hydrogen Bond	2.19	0.17		
	H- Acceptor	LYS129	H- Donor	Conventional Hydrogen Bond	2.59	1.80		
	H- Donor	ASP145	H- Acceptor	Conventional Hydrogen Bond	1.98	0.79		
	H- Donor		H- Acceptor	Conventional Hydrogen Bond	1.84	1.51		
	π –Orbitals are electron acceptors	ILE10	C-H bond is electron donor	Hydrophobic π - σ	3.64	-0.13		
Model 9						0.00	-17.36	906.60
	H- Acceptor	LYS278	H-Donor	Conventional Hydrogen Bond	2.17	0.38		
	π –Orbitals are electron donors		Alkyl group is electron acceptor	Hydrophobic π -Alkyl	3.75	-0.08		
	H- Acceptor	HIS119	H-Donor	Carbon Hydrogen Bond	2.99	0.63		
Model 3						0.00	-17.11	1.01×10^3
	H- Acceptor	VAL293	H-Donor	Conventional Hydrogen Bond	2.08	0.79		

	H-Donor	HIS295	H- Acceptor	Conventional Hydrogen Bond	2.93	0.96		
	π –Orbitals are electron acceptors	PRO292	C-H bond is electron donor	Hydrophobic π - σ	3.98	-0.08		
	π –Orbitals are electron donors		Alkyl group is electron acceptor	Hydrophobic π -Alkyl	4.24	-0.08		
Model 6						0.00	-16.02	1.55×10^3
	H- Acceptor	HIS295	H-Donor	Conventional Hydrogen Bond	2.20	0.17		
	π –Orbitals are electron acceptors		C-H bond is electron donor	Hydrophobic π - σ	3.79	-0.13		
	H-Donor	THR97	H- Acceptor	Conventional Hydrogen Bond	2.15	0.38		
	H-Donor		H- Acceptor	Conventional Hydrogen Bond	2.39	3.26		
	H- Acceptor	PRO294	H-Donor	Carbon Hydrogen Bond	3.48	-0.13		
Model 8						0.00	-14.77	2.57×10^3
	H- Acceptor	HIS296	H-Donor	Conventional Hydrogen Bond	2.06	0.79		
	H-Donor	VAL293	H- Acceptor	Conventional Hydrogen Bond	2.17	0.38		
	π –Orbitals are electron acceptors	PRO292	C-H bond is electron donor	Hydrophobic π - σ	3.66	-0.13		
	π –Orbitals are electron donors		Alkyl group is electron acceptor	Hydrophobic π -Alkyl	4.43	-0.08		
Model 2						0.00	-14.02	3.49×10^3
	H-Donor	HIS283	H- Acceptor	Conventional Hydrogen Bond	2.58	1.80		
	H-Donor	ALA282	H- Acceptor	Conventional Hydrogen Bond	2.12	0.38		
	π –Orbitals are electron donors	LEU281	Alkyl group is electron acceptor	Hydrophobic π -Alkyl	4.05	-0.08		
